# Global prevalence of occupational injuries among sanitation workers: a systematic review and meta-analysis

**DOI:** 10.3389/fpubh.2024.1425904

**Published:** 2024-10-03

**Authors:** Sina Temesgen Tolera, Tesfaye Gobena, Nega Assefa, Abraham Geremew

**Affiliations:** Haramaya University College of Health and Medical Sciences, Harar, Ethiopia

**Keywords:** burden, global, occupation, injuries, sanitary workers

## Abstract

**Background:**

In the sanitation sector, occupational injuries among sanitary workers (SWs) are prevalent due to hazardous working conditions and poor environmental surroundings. Despite the significant risks faced by these workers, the issue has received limited attention, and no comprehensive global meta-analysis on occupational injuries among sanitary workers has been conducted to date.

**Objective:**

In this study, we aimed to conduct a systematic review and meta-analysis of occupational injuries among sanitary workers globally from 2000 to 2023.

**Methods:**

The Preferred Reporting Items for Systematic Reviews and Meta-Analysis (PRISMA) guidelines were followed for the screening process, and the Population, Intervention, Comparison, Outcome and Study (PICOS) framework was to formulate search questions. Published articles from 2000 to 2023 were retrieved using various search engines. The keywords used were as follows: “Occupation Job Injuries” *OR “Work Injuries” *OR “Occupational Injuries” AND “Sanitary workers” (“Street sweepers” [SS] *OR “health facilities cleaners” [HCFC]) *OR “Solid waste collectors” [SWCs] *OR “Sewage workers” [STW] were used. Data analysis was performed using Stata Version 17MP. The overall effect size was calculated using the random-effects model combined with the restricted maximum likelihood (REML) approach, known as the Random-Effect REML Model. A 95% confidence interval (CI: 95%) was applied, and a *p*-value of less than 0.05 was considered statistically significant.

**Results:**

Studies were sourced from PubMed (*n* = 34), Medline (*n* = 39), Embase (*n* = 23), Global Health (*n* = 37), other databases (*n* = 54), and review studies (*n* = 10), resulting in a total of 197 studies. Of these, only 23 studies fully met the inclusion criteria. Among 8,138 sanitary workers (SWs), 4,469 (55%) were solid waste collectors (SWCs), 2,317 (28%) were street sweepers (SS), 1,144 (14%) were health facility cleaners (HCFC), and 208 (3%) were a combination of SS and SWCs. Globally, the pooled prevalence of occupational injuries among SWs was 36.49% (95%CI: 0.29–0.45). Specifically, 39.14% (95%CI: 0.24–0.53) prevalence was observed in high-income countries, while 35.22% (95%CI: 0.36–0.44) was reported in low-income countries. Year-by-year analysis showed a prevalence of 36.70% (95%CI:0.28–0.46) from 2001 to 2015 and 36.45% (95% CI:0.25–0.48) from 2016 to 2022. The overall heterogeneity of the studies was substantial, with an I-squared value of 90.03% and a heterogeneity index of 214.43 (*p* < 0.05), indicating statistically significant heterogeneity among the eligible studies.

**Conclusion:**

This systematic review and meta-analysis revealed that sanitation and hygiene workers face an increased risk of occupational injuries, largely due to insufficient attention to occupational safety and health services in their work environments. To mitigate these risks, the review recommends policy amendments, national regulations, and international initiatives aimed at improving occupational health and safety (OHS) services for these workers. These measures are crucial for reducing the prevalence of work-related injuries in the sanitation sector.

## Introduction

Sanitation and hygiene workers, often known as sanitation employees, play a crucial role in delivering safe sanitation services in homes, educational institutions, healthcare facilities, and other environments, thereby safeguarding public health (1). These workers are vital to global public health (2, 3), and their social wellbeing is equally important (4–6).

However, millions of sanitation workers in developing countries are forced to work in hazardous conditions that jeopardize their health and lives while undermining their dignity and human rights due to poor occupational health and safety (OHS) policies (5). They are usually among the most disadvantaged members of society, facing discrimination while working with minimal equipment and lacking strong legal protections (5). Economically, they often live in poverty, and (7) institutional neglect of OHS services results in frequent injuries (5).

In addition to these challenges, sanitation workers are often overlooked due to financial insecurity and social marginalization, including intergenerational discrimination (5, 8, 9). Moreover, their work is labor-intensive, and many of them face time pressures that contribute to mental stress. Studies highlight that factors such as high work rates, lack of control over the workload, limited access to assistance, and insufficient supervisory support exacerbate the mental strain experienced by these workers (10).

Numerous studies have highlighted the occupational risks faced by sanitary employees and healthcare workers, particularly from exposure to bodily fluids, blood, and infectious waste materials suspected of containing pathogens such as viruses, parasites, fungi, and bacteria. This includes cultures and stocks of pathogenic organisms from laboratory work, as well as waste from patients in isolation wards of hospitals (11–13).

Furthermore, studies have shown that garbage collectors, sewer workers, and SS are at risk of injuries and infections, such as hepatitis B and hepatitis C, as well as other workplace-related impairments (8). Sanitary workers (SWs) are frequently exposed to contaminated needles, sharps, and hazardous chemicals, especially those working in healthcare settings (2, 3). They may also come into contact with toxic substances, metal containers with chemical residue, and contaminants such as heavy metals (14).

Several studies have indicated that sanitation professionals experience psychological and mental health issues due to the demanding nature of their duties (15), as well as job instability and workplace violence related to their employment (16). These factors contribute to their dissatisfaction with daily work responsibilities (17).

Workplace-related issues manifest in various ways, including occupational illnesses, accidents, and injuries to the musculoskeletal system, leading to a range of adverse outcomes that impair job performance and efficiency. Consequently, sanitation workers often face wage losses due to reduced efficiency and absenteeism, and the costs of treatment and rehabilitation place a significant financial burden on society (18).

According to the Bureau of Labor Statistics, occupational injuries and illnesses are common causes of morbidity, disability, and poor quality of life among sanitation workers, with rates ranging from 56 to 90%. The most severe consequences stem from inadequate OHS practices, a lack of attention to OHS services, and unsafe working environments.

Reported injuries include abrasions, fractures, trauma, dislocation, bruises, burns, cuts, amputations and various diseases (2, 3, 19).

As a result, this study exclusively focused on the prevalence of work-related injuries, rather than other OHS outcomes, among sanitation personnel in the context of our systematic review and meta-analysis. “Occupational-related injuries” have long been recognized as one of the most serious consequences of workplace accidents. The severity of these injuries is an essential indicator for evaluating the outcomes of workplace accidents (20). Various metrics, such as the accident severity rate, the number of missed workdays, and the extent of damage to bodily parts, are commonly used to assess the severity of job-related injuries (21). The complexity of job-related injuries arises from a variety of contributing factors (22).

In this study, the term “prevalence of job-related injury” refers to the “self-reported occurrence of any physical harm to body tissues resulting from an accident or exposure to hazards within the 12 months preceding the incident” among sanitation workers. This definition aligns with previous studies and focuses on the “presence of work-related injury” experienced by sanitation employees within their working environments.”

Therefore, although it is not currently fully recognized or assessed internationally, collated information on occupational-related injuries among sanitation professionals is crucial for minimizing issues faced by these groups worldwide. As a result, the authors proposed four research questions (RQs_1–4_) to estimate the pooled prevalence of occupational injuries among SWs, which are included in the scope of this systematic review and meta-analysis.

*QR1*: What is the prevalence of occupational injuries among SWs worldwide?

*QR2*: What is the prevalence of occupational injuries among SWs in high- and low-income countries?

*QR3*: What is the pooled prevalence of occupational injuries among SWs for the periods 2000–2015 and 2016–2023?

*RQ4*: What is the pooled prevalence of occupational injuries among SWs after excluding the lowest and highest reported outcomes?

### What is already known on this topic?

Currently, the burden of occupational-related injuries is commonly observed among all employees and workers, particularly among SWs, due to their working conditions—unsafe, unhygienic, with greater exposure to numerous types of waste in various work settings such as municipalities, factories, commercial sectors, healthcare facilities, and plants. Moreover, many studies have indicated that SWs are exposed to numerous occupational hazards and accidents. They are also discriminated against, violated, and ignored by the rest of society. However, only a few studies have been conducted to quantify occupational-related injuries among these groups, which is why our current study aims to conduct systematic reviews and meta-analyses worldwide.

### What does this study add?

This systematic review and meta-analysis report provides critical evidence on the burden of occupational-related injuries among SWs globally. It examines differences between low-income and high-income countries, tracks trends over time, and compares various categories of SWs—information that has not been adequately reported yet. The study highlights the widespread prevalence of occupational injuries across different groups of SWs in their respective work environments.

### How might this study affect the research, practice, and/or policy?

The findings of this study offer important implications for both governmental and non-governmental organizations, such as the International Labour Organization, the World Health Organization, and similar initiatives, by urging the national ministries of health, labor, and social affairs to amend policies regarding this marginalized group. National ministries and associations should revise and incorporate comprehensive OHS regulations, policies, and guidelines specific to these workers. Furthermore, enforcement and monitoring of these amendments are essential to ensure effective implementation across all work sectors employing SWs.

## Methods

### Review protocols

Preferred Reporting Items for Systematic Reviews (PRISMA) revised criteria were applied to develop the flow diagram, which was adapted from (23). The systematic review questions and eligibility criteria were formulated based on the Population, Intervention, Comparison, Outcome, and Study Design (PICOS) framework, adapted from (24).

### Eligibility requirements

#### Inclusion criterion

- *Population* (P): Sanitary employees, such as SWCs, healthcare institution cleaners, and SS.
- *Intervention(I):* Occupational-related exposure.
- *Comparison (C):* Not applicable for this systematic review and meta-analysis.
- *Outcome(O):* Occupational, job-related, or any work-related injuries.
- *Study type(S):* Cross-sectional study.
- *Language:* Only papers published in the English language were included.
- *Article*s*/Studies:* Full-text articles and abstracts published in English with clear objectives, methodology, and quantitative outcomes were included.
- *Publication Year:* Studies published between 2000 and 2023.
- *Countries*: Both low-income and high-income countries were included.

#### Exclusion criteria

- *Population:* Office cleaners, hotel cleaners, and restaurant cleaners were excluded due to differences in their work type and employment characteristics.
- *Outcomes:* Studies focusing on non-occupational injuries were excluded.
- *Study Design:* Non-cross-sectional investigations were excluded, such as individual or cluster randomized controlled trials (RCTs). Non-randomized controlled trials (NTCs) include the following: quasi-RCTs, non-RCTs, controlled before-and-after studies, case–control studies, and cohort studies.
- *Language:* Papers published in non-English languages were excluded.
- *Articles/Studies:* Studies with unclear aims and methods were removed.
- *Publication:* Studies published before the year 2000 were excluded.

### Search engines and strategies

Four reviewers participated in this study. The systematic review and meta-analysis were conducted using a variety of search strategies across multiple databases, including PubMed, MEDLINE, Embase, Global Health electronic databases, Google Scholar, and other sources such as homepages. The studies were identified using EndNote (Version 20.4.1) and other methods for online search, focusing on databases such as PubMed, Medline, Embase, and Global Health.

The following keywords and MeSH terms were applied individually or in combination using Boolean logic operators (AND, OR):

- AND (Occupational-related injuries *OR Occupational injuries)
- AND (Street sweepers OR Health care facility cleaners *OR Solid waste collectors *OR Sewage workers OR Waste treatment workers)
- Countries (Developed *OR High-income countries *OR Developing OR Low-income countries)

These terms were used to refine the search to relevant studies on occupational injuries among SWs across various countries and income levels.

### Data screening

**Figure 1:**
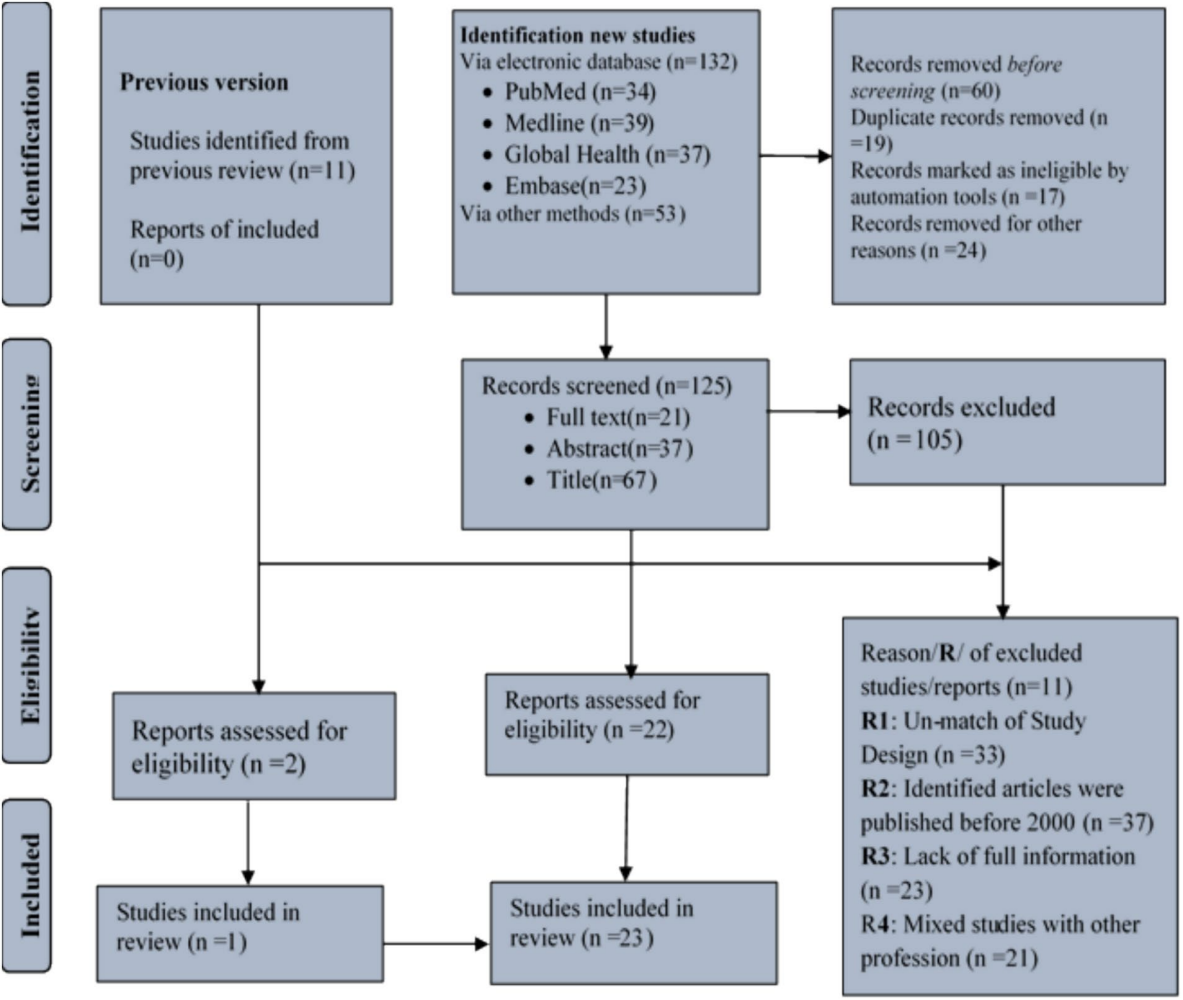
A flow diagram for systematic review adopted from PRISMA 2021.

Four reviewers participated in the data screening process. Titles and abstracts from the search results were filtered using Microsoft Excel, and full copies of the selected titles and abstracts were obtained for further review. The final results from the database search were managed using EndNote 20.4.1 reference management software and are summarized in Figure 1.

### Data extraction

Four reviewers were assigned to extract data using Microsoft Excel. A predefined extraction form, developed in an Excel spreadsheet, was utilized to capture the necessary information. The data sheet included details such as reference number, original authors, publication year, country, job categories, assessment instruments, and quality evaluation of each study.

### Data analysis

Three reviewers participated in the data analysis. Stata version 17/MP (StataCorp, Texas, United States) was used for the overall data analysis. The random-effects model and restricted maximum likelihood approaches were used to calculate the generic effect size (Random-Effect REML Model). The global prevalence of work-related injuries among sanitation workers was determined. Sub-analyses were conducted based on income level (low- and high-income countries), occupational categories, and year-to-year comparisons. Additionally, a sensitivity analysis was performed by excluding the three smallest and three largest extreme outcomes. A 95% confidence interval (CI) with a *p*-value of less than 0.05 was considered statistically significant for all analyses.

### Data synthesis

Four reviewers participated in synthesizing the data. Data synthesis was conducted based on the country, sanitary worker categories, and year. Eligible studies on occupational injuries among SWs were tallied, described, and summarized accordingly.

### Quality assessment

Four reviewers participated in the appraisal of the studies. The quality of the published studies was assessed using The Joanna Briggs Institute (JBI) Critical Appraisal Tools (Supplementary Table S2), which was specifically designed for cross-sectional studies, as adapted from (25). The tool consists of nine criteria, with each scored as (1) yes, (2) no, (3) unclear, or (4) not relevant. Studies were classified as having high publication bias (scores of 5), medium publication bias (scores of 5–7), or low publication bias (scores of 8–9). A visual funnel plot with a 95% confidence interval was used, and a *p*-value of less than 0.05 was considered statistically significant for the assessment of publication bias.

## Results

### Selection studies

A total of 197 (*n* = 197) studies were identified from the databases and other retrieved sources, including data and reports. After a thorough screening process, 23 studies were selected for inclusion in the final analysis (Figure 1).

### Study overview

A total of 23 (*n* = 23) studies were deemed eligible for the systematic review and meta-analysis, focusing on the pooled prevalence of occupational-related injuries among SWs across globally (Table 1).

**Table 1:** Authors, year of study, countries, design used, tool used, and categories of SWs.

Ref. no	Author/s	Year	Countries	Design used	Tool used	Category (*N* = 8,138)	Publication bias
(26)	Eskezia et al.	2016	Ethiopia	CS	Questionnaire	SWCs (*n* = 379)	Low
(37)	Alamgir and Shicheng	2008	Columbia	CS	Questionnaires	HCFC (*n* = 145)	Low
(49)	Sangkham et al.	2021	Thailand	CS	Questionnaires	SWCs (*n* = 107)	Medium
(27)	Mamuya and Badi	2019	Tanzania	CS	Questionnaires	SWCs (*n* = 354)	Low
(45)	Rahma et al.	2009	Egypt	CS	Questionnaires	SS + SWCs (*n* = 70)	Medium
(28)	Ephraim et al.	2021	Ghana	CS	Questionnaires	SWCs (*n* = 358)	Low
(29)	Bogale et al.	2014	Ethiopia	CS	Questionnaires	SWCs (*n* = 876)	Low
(38)	Salwe et al.	2011	Texas, USA	CS	Questionnaire	HCFC (*n* = 106)	Medium
(30)	Steven	2016	Zimbabwe	CS	Questionnaires	SWCs (*n* = 589)	Medium
(46)	Ewis et al.	2013	Egypt	CS/CG	Questionnaire	SS + SWCs (*n* = 138)	Low
(31)	Melaku and Tiruneh	2020	Ethiopia	CS	Questionnaires	SWCs (*n* = 576)	Low
(39)	Alwali et al.	2021	Palestinian	CS	Questionnaires	HCFC (*n* = 104)	Medium
(40)	Kaweti and Abegaz	2016	Ethiopia	CS	Questionnaires	HCFC (*n* = 100)	Medium
(41)	Saadeh et al.	2020	Jordan	CS	Questionnaires	HCFC (*n* = 144)	Low
(42)	Shiao et al.	2001	Taiwan	CS	Questionnaires	HCFC (*n* = 147)	Medium
(32)	Temesgen et al.	2022	Ethiopia	CS	Questionnaires	SWC (*n* = 381)	Low
(33)	Marew	2015	Ethiopia	CS	Questionnaires	SWCs (*n* = 635)	Medium
(51)	Coelho	2012	Brazil	CS	Questionnaires	SWCs (*n* = 97)	Medium
(34)	Uhunamure et al.	2021	South Africa	CS	Questionnaires	SWCs (*n* = 114)	Low
(35)	Pinho and Neves	2010	Brazil	CS	Questionnaires	SWCs (*n* = 36)	High
(43)	Johnson and John	2020	Nigeria	CS	Questionnaires	SS (*n* = 150)	Medium
(44)	Weng et al.	2022	China	CS	Questionnaires	SS (*n* = 2,167)	Low
(36)	Ayzohbel	2021	Ethiopia	CS	Questionnaires	HCFC (*n* = 398)	Medium

CS, Cross-sectional study; CG, group control; HCFC, healthcare facility cleaners; SCWs, solid waste collectors; SS, street sweepers.

### Eligible countries

A total of 15 countries from across the globe were eligible for inclusion in the current review. Of these, seven countries were classified as developed countries, while eight of them were from developing countries. The top three countries with the highest number of reviewed studies were Ethiopia (*n* = 6 studies), Egypt (*n* = 2 studies), and Brazil (*n* = 2 studies), as detailed in Supplementary Table S2.

### Eligible population

The total population of SWs eligible for this systematic review and meta-analysis was 8,138. Among them, municipality SWCs accounted for 4,469 (55%), SSs made up 2,317 (28%), HCFCs represented 1,144 (14%), and street SSs combined with municipality SWCs comprised 208 (3%) of the total population (Supplementary Table S2).

### Pooled prevalence of injuries

#### By regions

**Figure 2:**
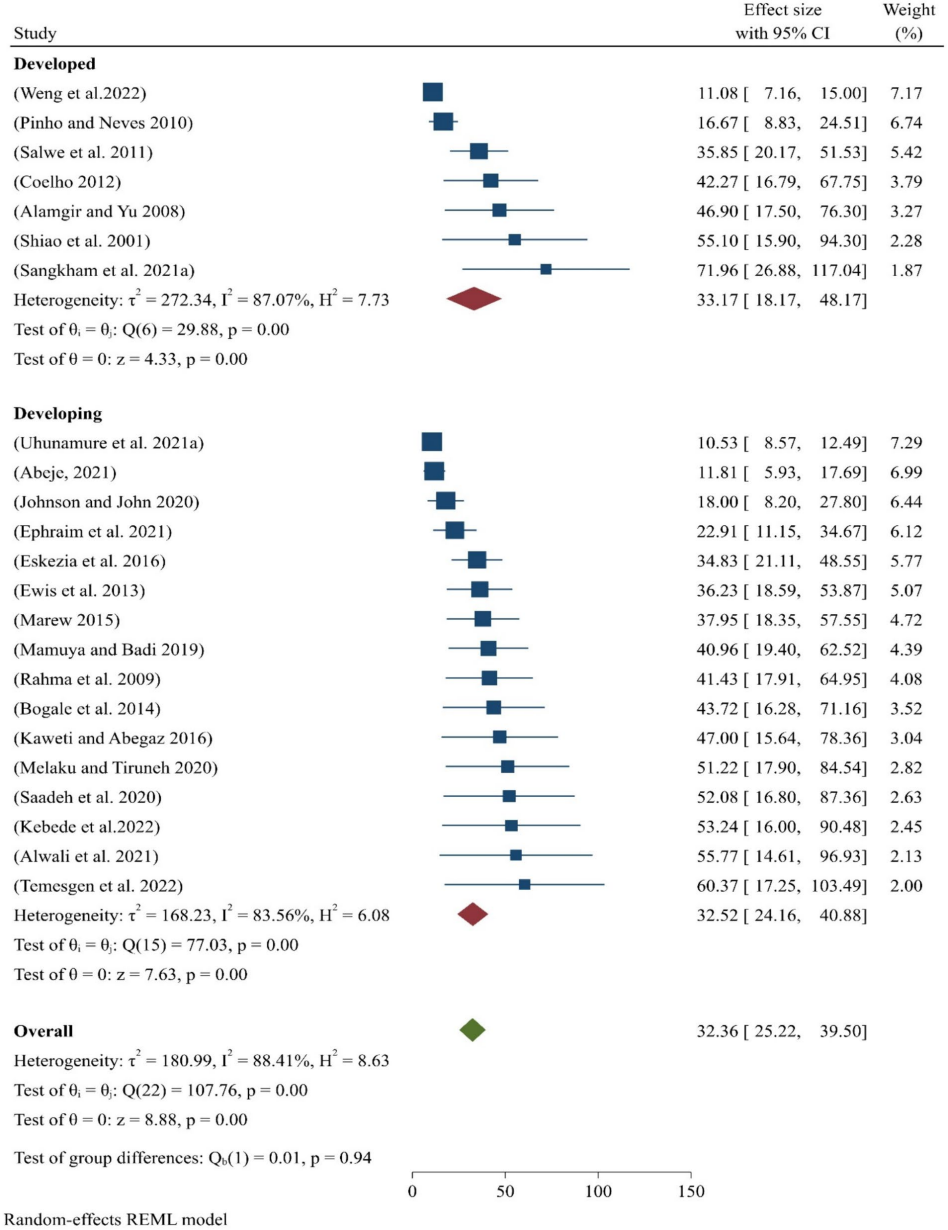
Pooled prevalence of occupational injuries among SWs worldwide.

The global pooled prevalence of occupational-related injuries among all SWs was 32.36 (95% CI:25.22–39.50), which is significantly high due to the challenging working conditions faced by these workers. When analyzed by regional categories, the prevalence in high-income countries was 33.17% (95% CI:18.17–48.17), while in low-income countries, it was 32.52% (95% CI:24.16–40.88) (Figure 2).

#### By subgroups

**Figure 3:**
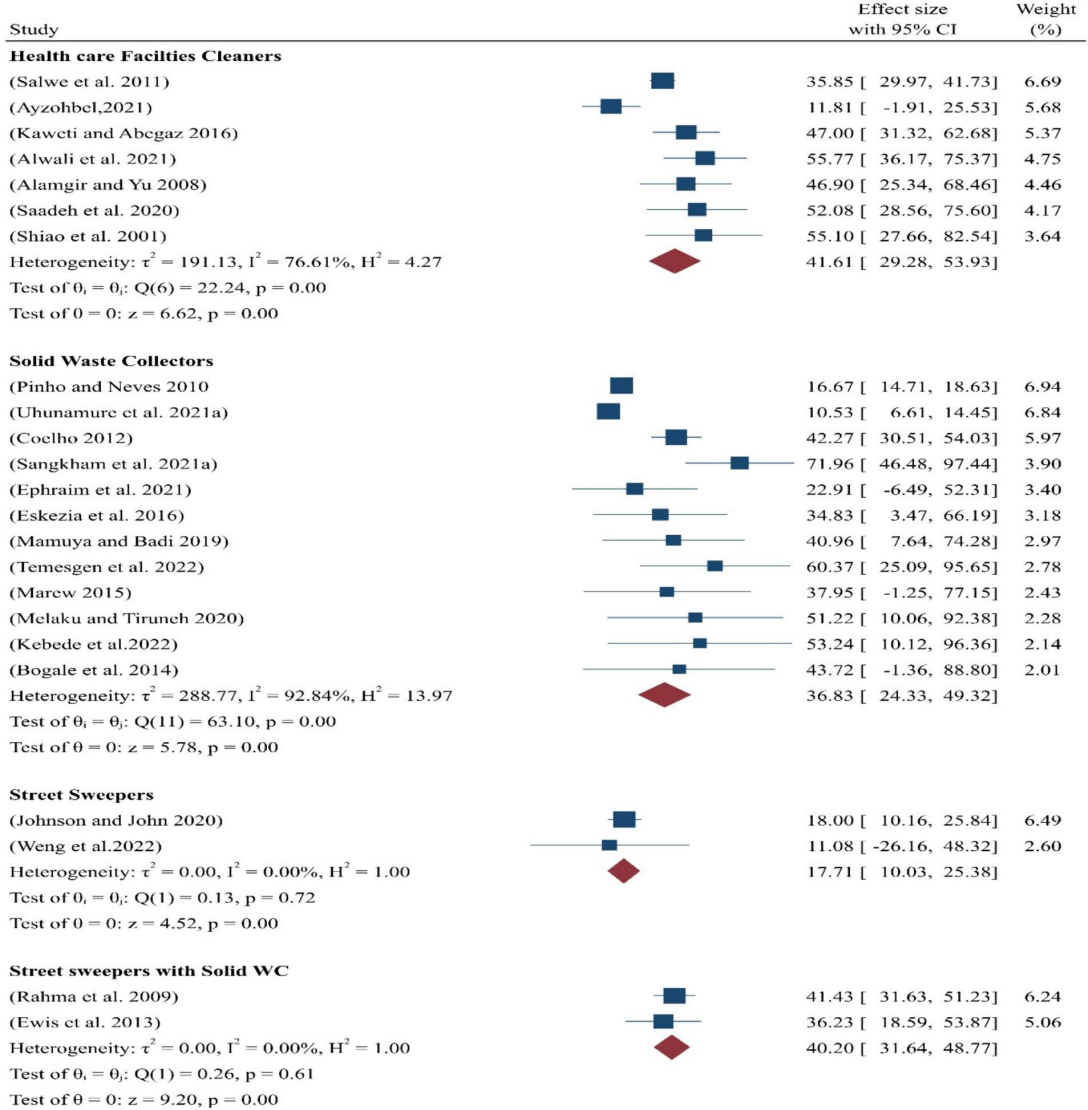
Pooled prevalence of occupational injuries among the subgroups of SWs.

A meta-analysis was also conducted for the subgroup analysis of different categories of SWs. The findings revealed that the pooled prevalence of occupational-related injuries was highest among healthcare facility cleaners, at 41.61% (95% CI:29–54), compared to other categories of sanitary employees (Figure 3).

#### By year-by-year

**Figure 4:**
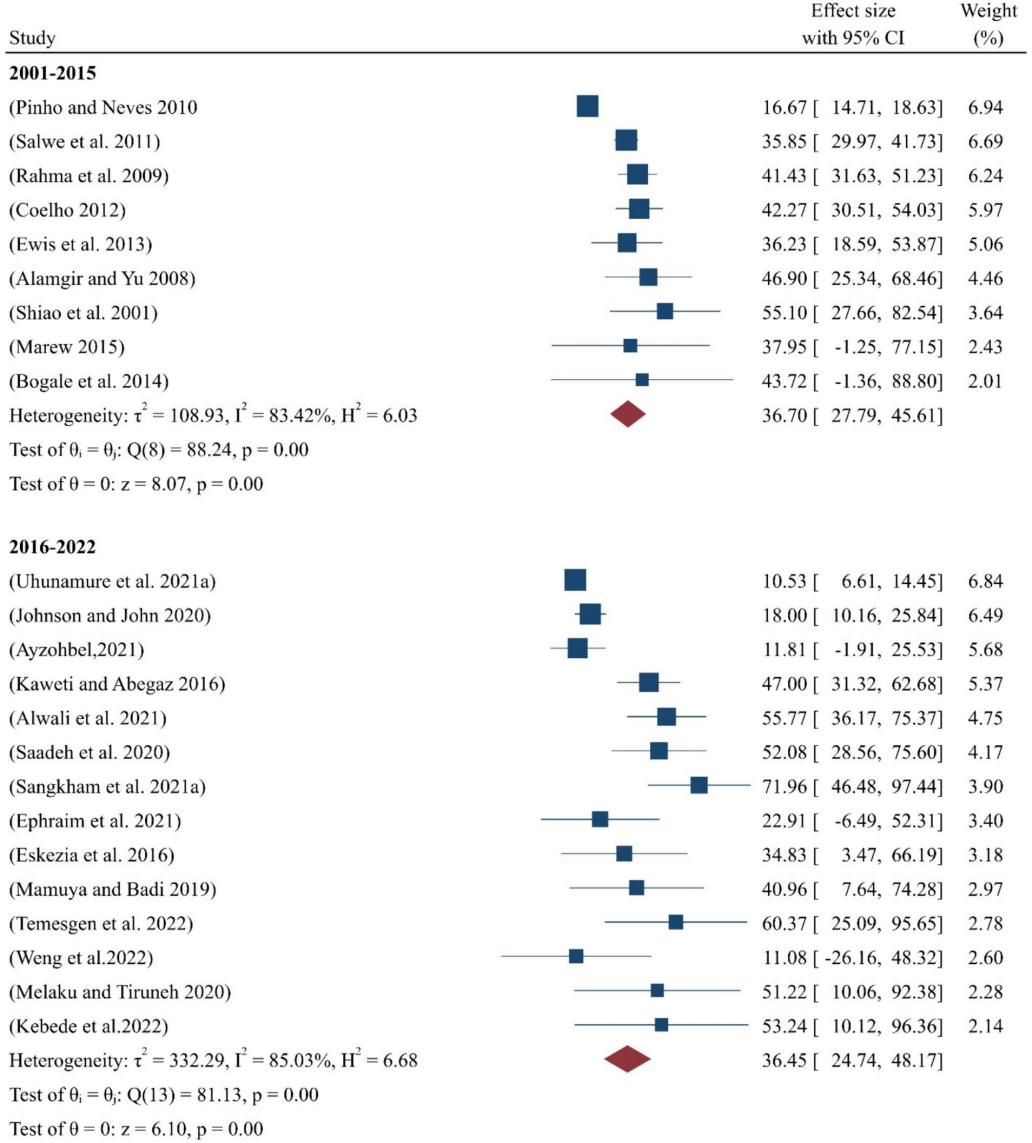
Pooled prevalence of occupational injuries among SWs by year.

According to the years sub-analysis, the pooled prevalence of occupational-related injuries among SWs from 2000 to 2015 was 36.70% (95%CI:28–46), while for the period between 2016 and 2022, it was 36.45% (95%CI:25–48) (Figure 4).

#### Sensitivity analysis

**Figure 5:**
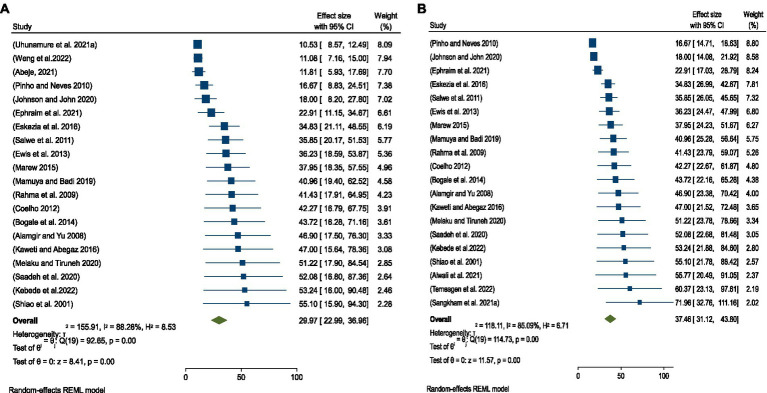
**(A)** Sensitivity after excluding the three smallest outcomes among SWs. **(B)** Sensitivity analysis after excluding the three largest outcomes among SWs.

The sensitivity analysis was conducted to examine the variation in outcomes among the eligible studies. After excluding the three smallest outcomes, the pooled prevalence of occupationally associated injuries among SWs was 37.46% (95%CI:31.12–43.80) (Figure 5A). Conversely, after excluding the three greatest outcomes, the pooled prevalence was 29.97% (95%CI:22.99–36.96) (Figure 5B).

#### Publication bias

**Figure 6:**
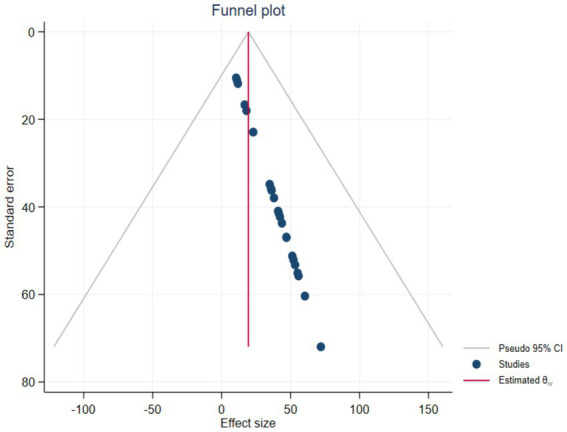
Publication bias among eligible studies (2000–2023) for systematic review and meta-analysis.

The overall quality of the study considered in this systematic review and meta-analysis was 73.43%, as indicated in additional material (Supplementary Table S2). The meta-regression shows that the scatter distribution across the funnel plot is not uniform, indicating the potential presence of publication bias among the eligible studies (Figure 6).

## Discussion

In the current systematic review and meta-analysis, a total of 197 studies were identified through various databases and other sources. Approximately 24 studies were removed due to unclear or mixed design, and 60 studies were excluded prior to screening because they were duplicate records. Additionally, 17 studies were excluded as they did not meet the review criteria. Afterward, the new studies and those from an earlier review version were further filtered, leaving 125 new studies and 11 studies from the previous review. A total of 114 studies were then excluded due to non-compliance with the study design, incorrect publication year, insufficient information, and because the studies combined sanitary staff with other professions.

Using data from 23 studies, the global pooled prevalence of occupational injuries among SWs was determined (Figure 1). Of these studies, seven of them were from high-income countries, while the remaining eight studies were from low-income countries. A significant portion of the research was conducted in Ethiopia, followed by Egypt and Brazil. (Supplementary Table S1). In terms of population distribution, 55% of the SWs were SWCs, which were drawn from 11 studies (26–36). Similarly, healthcare facility cleaners accounted for 14% of the workers, based on seven studies (36–42). Two studies (43, 44) focused on SS, who made up approximately 28% of the SWs. The remaining two studies (45, 46), which combined SS and SWCs, accounted for 3% of the population (Supplementary Table S1).

The global pooled prevalence of occupational injuries among all SWs was found to be 32.36% (Figure 2). This high rate may be attributed to the lack of attention to occupational safety and health services in the workplace. Many studies reported insufficient focus on occupational safety and health services (26–36), as well as a lack of OHS training and supervision (36–46). Additionally, the lack of personal protective equipment (43, 44) further exacerbates the risks in these settings for SWs.

In contrast, the current finding of 32.36% is lower than the previously reported prevalence of 44.66% (47). This difference may be attributed to the fact that the earlier study was conducted solely in a low-income country, while the present analysis was conducted on a global scale. To assess the variability of research collected from both high- and low-income nations, a heterogeneity analysis was performed. The overall I-squared heterogeneity in this study was 88.41% (*p* < 0.05), demonstrating statistically significant heterogeneity, as it falls within the range of 75–100% (48).

Moreover, the pooled prevalence of occupational-related injuries among all sanitary employees differed across high- and low-income countries. In seven high-income countries, the pooled prevalence was 33.17% (Figure 2), reflecting a statistically significant with working conditions. The prevalence ranged from a minimum of 16.7% in Brazil (35) to a maximum of 72% in Thailand (49). A heterogeneity analysis was conducted to understand the variation in studies from high-income nations, resulting in an I-squared heterogeneity of 87.07%, which, according to Higgins’ interpretation (*p* < 0.05), indicates considerable heterogeneity, as it falls within the range of 75–100% (48).

Similarly, the aggregated or pooled prevalence of occupational-related injuries among all sanitary employees in the eight low-income countries was 32.52% (Figure 2). The lowest prevalence was 10.5% in South Africa, while the highest prevalence was 60.4% in Ethiopia.

According to this research, the prevalence of occupational-related injuries varies significantly between South Africa and Ethiopia, with the rate in Ethiopia being six times higher than in South Africa. This disparity could be due to several factors, including SWs’ perceptions of occupational risks, differences in organizational commitment and support, and public acceptability, each of which is a critical predictor of occupational injury occurrence. Additionally, a heterogeneity analysis was conducted to understand the diversity of research from low-income countries. The overall heterogeneity, as measured by I-squared, was 87.07% (*p* < 0.05), indicating considerable heterogeneity according to Higgins’ interpretation (48). This high level of variability highlights the different conditions and challenges faced by SWs in low-income nations.

Moreover, in comparing low-income and high-income countries, the pooled prevalence of occupational-related injuries among SWs in high-income countries was slightly higher than in low-income countries (Figure 2). This difference may be attributed to the unequal proportions of studies collected from these countries. Fewer studies were found in high-income nations, while a larger number were discovered in low-income countries, leading to greater variability in effect size for low-income countries. However, this does not necessarily imply that SWs in high-income countries face higher occupational risks. In fact, the occupational hazards in low-income countries may have a more detrimental impact on SWs’ motivation and identification with their profession. In low-income countries, SWs often lack regular training and access to necessary equipment, which leads to low job satisfaction. This, in turn, results in negative physical health outcomes, commonly recognized as occupational injuries or physical harm (50).

Furthermore, a sub-analysis was conducted to explore the variability and heterogeneity among different groups of SWs based on their work environments. The pooled prevalence of occupational-related injuries was highest among healthcare facility cleaners, at 41.61%, as reported in seven studies (36–42) (Figure 3). The heterogeneity for these studies, with an I-squared value of 76.61% (*p* < 0.05), indicates significant variability, as it falls between 75 and 100%, as interpreted by (48).

Similarly, the pooled prevalence of occupational-related injuries among SWCs was 36.83%, which is based on data from 11 studies (26–36).

This result demonstrates a statistically significant association with the working conditions of SWCs.

In the third sub-analysis category, the pooled prevalence of occupational injuries among a group of SS and municipality SWCs was 40.20%, obtained from two studies (45, 46). However, this result was not statistically significant with regard to their working conditions. Finally, the pooled prevalence of occupational injuries among SS was the lowest, at 14.0%, based on two studies (43, 44), and their working conditions were also not found to be statistically significant (Figure 3).

Furthermore, the year-by-year sub-analysis indicated that the pooled prevalence of occupational injuries among SWs was 36.70% for the period between 2001 and 2015 (Figure 4), based on eight studies conducted worldwide (29, 33, 35, 38, 42, 45, 46, 51). As shown in this figure, the overall heterogeneity of the studies from 2001 to 2015 had an I-squared value of 83.42% (*p* < 0.05), indicating significant heterogeneity, as it falls within the range of 75–100%, according to (48) the interpretation.

Similarly, the pooled prevalence of occupational injuries among SWs from 2016 to 2022 was 36.45%, based on 15 studies (26–28, 30–32, 34, 36, 37, 39–41, 43, 44, 49). The overall heterogeneity for the studies from this period was I-squared 85.03% (*p* < 0.05), which also indicated significant heterogeneity within this range, as stated by (48). The results of this meta-analysis, conducted through year-by-year sub-analysis, show that the pooled prevalence of occupational injuries among SWs remained nearly identical between the two periods, 2001–2015 and 2016–2022.

In this systematic review and meta-analysis, a sensitivity analysis was performed by separately removing the three smallest and three largest extreme outcomes of occupational injuries to assess the impact on the overall pooled prevalence. After excluding the three smallest outcomes, the pooled prevalence of occupational injuries among SWs worldwide increased to 37.46% (Figure 5A). This is higher compared to the original pooled prevalence of 32.36% (Figure 2). Similarly, after removing the three largest extreme outcomes, the pooled prevalence decreased to 29.97% (Figure 5B), which is lower than the original pooled prevalence (Figure 2).

These findings suggest that the inclusion of extreme values significantly affects the pooled prevalence of occupational injuries among SWs. The difference between the original prevalence and the adjusted values after excluding the extremes could indicate the presence of publication bias, as extreme outcomes may disproportionately influence the aggregated results.

Regarding publication bias, after screening, 23 studies were evaluated using nine criteria from the Joanna Briggs Institute (JBI) checklist (Supplementary Table S2). According to the criteria, over half of the studies had low publication bias, with approximately 45% showing moderate publication bias. Only one study had considerable publication bias (Table 1). Based on the JBI criteria, the 23 studies should have achieved approximately 207 points. However, only 152 points were met, accounting for 73.43% (Supplementary Table S2).

In addition, the meta-analysis revealed unequal scatter distribution across the funnel plot. Of the 23 studies, only five were fully within the funnel, and these were not evenly dispersed along the vertical line. This unequal distribution suggests the presence of publication bias among the eligible studies (Figure 6). The critical appraisal further highlighted selection bias, as many studies did not adequately recruit participants, address the target population, or use reliable procedures to assess occupational injuries.

The inclusion and exclusion criteria, as well as the methods for selecting personnel involved in workplace cleaning, were not clearly outlined in most studies. This type of publication bias may stem from selection bias, which is a notable concern in this systematic review and meta-analysis. Selection bias presents a significant issue, largely due to random variations and the lower methodological quality of smaller studies.

## Limitations

This meta-analysis and systematic review have a few limitations to be considered, similar to those observed in other meta-analyses and systematic reviews. These limitations include heterogeneity in the research settings, demographics, study designs, exposure assessments, and outcome evaluations. Additionally, many of the publications reviewed did not adequately describe the demographic characteristics of the sanitation workers studied. There is also the possibility that the sanitation workers were not uniformly exposed to human feces, wastewater, solid waste, or hospital hazards. Furthermore, almost all the included studies employed a cross-sectional design, which may introduce selection and information bias during the sampling process, with confounding factors being another limitation of this design.

## Conclusion

Current evidence reveals that the highest pooled prevalence of injuries among SWs was found worldwide. The outcome can be attributed to unsafe hygiene practices, poor working environments; poor and little attention to OHS protocols in institutions, and a lack of institutional or organizational commitment. As a result of this research, it is expected that significant changes in governmental policies and actions will be implemented to mitigate these risks, particularly in middle- and low-income countries.
