# Supplementary material for: Global prevalence of occupational injuries among sanitation workers: a systematic review and meta-analysis
Source: Front Public Health. 2024 Oct 3;12:1425904. doi: 10.3389/fpubh.2024.1425904 (PMC11483865; doi:10.3389/fpubh.2024.1425904)
Supplement: Supplementary file 1 [file Table_1.docx]

**Supplementary Table 1(Sup. Table 1)**

Sup. Table 1. Number of studies and type of sanitary workers found from developed countries and developing countries

| **S.no** | **Countries** | **Region** | **No of Studies** | **Category of SWs** | **No of SWs** |
| --- | --- | --- | --- | --- | --- |
| 1 | Brazil | Developed | 2 | SWCs | 135 |
| 2 | Columbia | Developed | 1 | HCFC | 145 |
| 3 | Egypt | Developing | 2 | SS +SWCs | 208 |
| 4 | Ethiopia | Developing | 7 | SWCs  HCFCs | 2847  498 |
| 5 | Ghana | Developing | 1 | SWCs | 358 |
| 6 | Jordan | Developed | 1 | HCFC | 144 |
| 7 | Thailand | Developed | 1 | SWCs | 107 |
| 8 | Tanzania | Developing | 1 | SWCs | 354 |
| 9 | Texas, USA | Developed | 1 | HCFCs | 106 |
| 10 | Zimbabwe | Developing | 1 | SWCs | 589 |
| 11 | Palestinian | Developing | 1 | HCFCs | 104 |
| 12 | Taiwan | Developed | 1 | HCFCs | 147 |
| 13 | South Africa | Developing | 1 | SWCs | 114 |
| 14 | Nigeria | Developing | 1 | SS | 150 |
| 15 | China | Developed | 1 | SS | 2167 |
|  | Total | Developed=7 | 8 | SWCS | 4469(55%) |
|  |  | Developing=8 | 15 | HCFCs | 1144(14%) |
|  |  |  |  | SS | 2317(28%) |
|  |  |  |  | SS+SWCs | 208(3%) |
|  |  | Grand | 23 | - | 8138(100%) |
