# Supplementary material for: Global prevalence of occupational injuries among sanitation workers: a systematic review and meta-analysis
Source: Front Public Health. 2024 Oct 3;12:1425904. doi: 10.3389/fpubh.2024.1425904 (PMC11483865; doi:10.3389/fpubh.2024.1425904)
Supplement: Supplementary file 2 [file Table_2.docx]

**Supplementary Table 2 (Sup. Table 2)**

Sup. Table 2: JBI Critical Appraisal Checklist for studies eligible in Systematic Review and Meta-analysis

| **Statement of JBI for Identified Studies (n=23)** | **Total Yes (=X/23)** | **%** |
| --- | --- | --- |
| 1. Was the sample frame appropriate to address the target population? | 15 | 64 |
| 1. Were study participants sampled in an appropriate way? | 12 | 66.7 |
| 1. Was the sample size adequate? | 17 | 54.2 |
| 1. Were the study subjects and the setting described in detail? | 17 | 79.2 |
| 1. Was the data analysis conducted with sufficient coverage of the identified sample? | 17 | 79.2 |
| 1. Were valid methods used for the identification of the condition? | 17 | 79.2 |
| 1. Was the condition measured in a standard, reliable way for all participants? | 18 | 75.0 |
| 1. Was there appropriate statistical analysis? | 19 | 83.3 |
| 1. Was the response rate adequate, and if not, was the low response rate managed appropriately? | 19 | 87.5 |
| **Overall evaluation** | 152 | 73.43% |
